# Quasi-Randomized Trial of Effects of Perioperative Oral Hygiene Instruction on Inpatients with Heart Diseases Using a Behavioral Six-Step Method

**DOI:** 10.3390/ijerph16214252

**Published:** 2019-11-01

**Authors:** Chie Omori, Daisuke Ekuni, Yumiko Ohbayashi, Minoru Miyake, Manabu Morita

**Affiliations:** 1 Department of Preventive Dentistry, Okayama University graduate School of Medicine, Dentistry and Pharmaceutical Sciences, Okayama 700-8558, Japan; pspc6q1h@s.okayama-u.ac.jp (C.O.); mmorita@md.okayama-u.ac.jp (M.M.); 2 Department of Oral Health Sciences, Baika Women’s University, Ibaraki 567-8578, Japan; 3 Department of Oral and Maxillofacial Surgery, Kagawa University Hospital, Kagawa 761-0793, Japan; yumiko@med.kagawa-u.ac.jp (Y.O.); dentmm@med.kagawa-u.ac.jp (M.M.)

**Keywords:** oral hygiene instruction, perioperative period, self-efficacy, six-step method

## Abstract

The assessor-blinded, parallel-design, quasi-randomized study (alternating allocation) aimed to determine the effects of the six-step method on postoperative numbers of oral bacteria, periodontal status, and atrial fibrillation (AF) among inpatients with heart diseases and periodontitis. Seventy inpatients who received preoperative periodontal treatment were quasi-randomly assigned to intervention and control groups at University Hospital. The intervention group received intensive oral hygiene instruction using a six-step method for 15 minutes per week and the control group received routine oral hygiene instruction. Significantly fewer oral bacteria were identified on the tongue at discharge compared with baseline in the intervention than the control group (ANCOVA) (large effect size, *p* = 0.02). Changes in scores for self-efficacy, plaque scores, probed pocket depth, and bleeding on probing between baseline and discharge were significantly greater in the intervention, than in the control group (*p* < 0.05). The period of postoperative AF (days) was significantly shorter in the intervention, than in the control group (*p* = 0.019). In conclusion, oral hygiene instruction using the six-step method decreased the numbers of oral bacteria on the tongue and improved self-efficacy, oral health behaviors, oral hygiene status, periodontal status, and period of postoperative AF among inpatients with periodontitis and heart diseases.

## 1. Introduction

Atrial fibrillation (AF) is associated with increased morbidity and mortality [1] and it remains the most common complication after cardiac surgery, affecting 20%–50% of patients [2,3,4,5]. AF is associated with other cardiovascular diseases and increasing evidence has shown links between AF and local as well as systemic inflammation, such as obesity and rheumatoid arthritis [6,7]. However, the risks associated with postoperative AF are not fully understood [8,9].

Periodontitis is an inflammatory disease of the supporting structures of the teeth. As periodontitis is a possible trigger of chronic inflammation, recent studies suggest a link between periodontitis and heart diseases [10,11,12,13]. Recent studies on animal models [14] and humans [15] have suggested an association between AF and periodontitis. Thus, reducing infection with oral bacteria and inflammation is very important for the management of both AF and periodontitis.

Postoperative, professional oral hygiene care can reduce the number of oral bacteria on the tongue [16], the numbers of oropharyngeal bacteria [17], and complications such as pneumonia [18]. On the other hand, self-care as well as professional care are needed to improve postoperative oral hygiene status, which contributes to complications. Thus, effective oral hygiene instruction is desirable to improve self-care among inpatients. 

The six-step method can be an effective oral hygiene instruction [19,20]. It is based on the self-efficacy theory (self-confidence that one can take action necessary to lead to a specific result) [21] and employs a behavioral science approach that consists of six steps [22]. In dentistry, the self-efficacy scale for self-care (SESS) was developed for dental outpatients to evaluate self-efficacy. High SESS was correlated with better oral hygiene and periodontal health [19,23]. The six-step method was more effective for enhancing self-efficacy and behavioral change in oral hygiene than traditional oral hygiene instruction alone in outpatients [20]. However, whether this method is actually effective among inpatients is unknown. 

For inpatients, it is very important to improve oral hygiene status including that of the tongue. Previous studies showed that heavier tongue coating precipitation increased the pneumonia incidence rate [24] and prolonged hospitalization days [25]. Furthermore, oral bacteria on the tongue were associated with periodontal status and sub-gingival microbiome [26,27]. Thus, we focused on oral bacteria on the tongue. We postulated that the six-step method would improve self-efficacy and oral hygiene status, including tongue, and then contribute to periodontal status among inpatients with heart diseases. Therefore, the present study aimed to determine the postoperative effects of the six-step method on the numbers of oral bacteria on the tongue, periodontal status, and AF among inpatients with heart diseases.

## 2. Materials and Methods 

### 2.1. Design

This assessor-blinded, parallel-design, single-center, quasi-randomized study (alternating allocation) of inpatients at Kagawa University Hospital, Japan, between June 2016 and July 2017 followed Consolidated Standards of Reporting Trials (CONSORT) guidelines and was approved by the Ethics Committee at the Medical Faculty of Kagawa University (No. 28-026). All enrolled inpatients provided written informed consent to participate in this study. Eligibility criteria did not change between before and after the trial, which was registered under Current Controlled Trials in UMIN-ICDR (UMIN000031047).

### 2.2. Blinding

Study personnel, including the periodontal examiners and the investigator responsible for the data analysis, were blinded to intervention assignments. The patients were not blinded.

### 2.3. Sample Size Calculation

Sample size was estimated assuming a 1.0 × 107 (SD, 1.3 × 107) cfu/mL or greater reduction in the numbers of oral bacteria on the tongue in the intervention compared with the control group [16]. Based on the data, we determined that 28 inpatients per group would be necessary to provide 80% power with an alpha of 0.05 in two-tailed and unpaired t-tests. Assuming an attrition rate of 18%, the planned sample size was 70 participants (35 per group).

### 2.4. Participants

Participants were recruited between June 2016 and July 2017 at the Department of Cardiovascular Surgery, Kagawa University Hospital. The inclusion criteria were heart diseases and admission to hospital for surgery. Exclusion criteria included inappropriate status for the trials, such as failure of written informed consent to participate in the study at the Department of Dentistry, receiving periodontal treatment and using systemic antibiotics during the six months before the trial started [28] or not having any teeth.

### 2.5. Intervention

All patients received mechanical tooth cleaning, scaling without using local anesthetic, and removal of the tongue coating at the dental chair of the Dentistry by dental hygienists in a single visit before operation. After the preoperative treatment, the patients were quasi-randomly and alternately assigned to intervention and control groups (1:1) [29].

Patients in the intervention group received intensive oral hygiene instruction by the six-step method 15 minutes per week [19,23]. The six-step method comprised identification of the problem, establishment of commitment and confidence, increased awareness of behavior, development and implement of an action plan, evaluation of the plan, maintenance of change, and the prevention of relapse. This summarizes how to apply the self-efficacy theory in health education [19,20,23,30].

The previous six-step method [20] was modified for perioperative inpatients as follows.

• STEP 1: Identification of the problem

Knowledge, belief, and barriers to self-care were clarified by a dental hygienist in interviews. The information was obtained using the following questions: (i) How many times do you brush your teeth?; (ii) Do you have regular dental check-ups?; (iii) Do you use interdental brushes?; (iv) Do you clean your tongue?; (v) Have you previously tried to change your oral hygiene behavior?; (vi) What stops you from changing your beliefs or what are your major barriers to change? We also disclosed dental plaque accumulation using Red Cote Liquid (Sunstar Americas Inc. Schaumburg, IL, USA) so that each patient could determine areas that they were not brushing appropriately. The patients thus identified their problems.

• STEP 2: Creation of commitment and confidence

The clinical interview and counseling were expanded. The patients learned the importance of maintaining good perioperative oral hygiene status. A dental hygienist encouraged the patients to confirm their intention and to promote motivation.

• STEP 3: Increase awareness of behavior

Patient awareness of their own behavior was increased through self-monitoring. A dental hygienist instructed the patients to maintain a diary regarding toothbrushing and the use of interdental brushes to monitor their accomplishments and identify barriers to changes in behavior.

• STEP 4: Development and implementation of an action plan

Short-term action plans established according to the principle of gradualism were based on the skill, behavior, and oral hygiene status of each patient. These plans were concrete, realistic, achievable, and included “brush teeth three times each day”, “use interdental brushes daily”, or “clean the tongue every day”. Patients set goals that they could achieve by the time of the next interview.

• STEP 5: Evaluation of the plan

Whether or not the patient implemented the action plan was evaluated. Successful experiences were acknowledged and supported. When the plan succeeded, the success experience was acknowledged. The dental hygienist praised improvements in the oral hygiene status of the patients, even if quite small. Failure was attributed to failure of the plan, and a new achievable plan was established.

• STEP 6: Maintenance of change and prevention of relapse

Some inpatients had perioperative high-risk situations that resulted in relapse; for instance, postoperative poor physical status, a sink located far from their hospital bed, or limited range of hand movement due to intravenous drips. Thus, it was important for the dental hygienist to safeguard and reinforce the new behaviors to help and encourage the patients.

Patients in the control group received routine oral hygiene instruction for 15 minutes per week. The technical guidance [31] of brushing was main part of routine oral hygiene instruction in the control group. The dental hygienists instructed mainly the Bass method and how to use interdental brushes according to the patients’ oral condition. The oral hygiene instruction was standardized and assessed among dental hygienists sharing information of each patient.

A typical content of instruction in each group is shown in the Table 1. Instructions were started in both groups from postoperative day (POD) 3. Dental hygienists provided routine postoperative oral hygiene care, mechanical tooth cleaning, and tongue coating removal to both groups. Dental hygienists checked the adherence of patients to interventions.

### 2.6. Outcome Assessment

The primary study outcome was a change in the numbers of oral bacteria on the tongue between baseline and discharge. The sampling at discharge was performed 1 week after the final postoperative care by the dental hygienists. Secondary outcomes included changes in oral hygiene status, periodontal parameters, tongue coating scores, self-efficacy scores, and the incidence of postoperative AF.

### 2.7. Measurements of Oral Bacteria on the Tongue

Dental hygienists collected coatings from the surface of the tongue at the middle of the dorsum linguae for oral bacteria counts using a cotton swab with gentle pressure according to the manufacturer’s procedure [16,23]. The numbers of oral bacteria in the samples were immediately measured using a simple, portable bacterial counter (Panasonic Healthcare Co. Ltd., Tokyo, Japan) [32] and are presented as colony-forming units (cfu/mL). After the training, to assess the examiner agreement, the numbers of oral bacteria on the tongue were recorded at the same time in each volunteer on preliminary calibration. The errors of measurement were <6.4%.

### 2.8. Oral Examination

O’Leary’s Plaque Control Record (PCR) [33] was determined after staining with Red Cote Liquid (Sunstar) and recorded with respect to plaque location (mesial, distal, buccal, and lingual) relative to the gingival margin around each tooth. A total of 30 probed pocket depths (PPDs) were probed at mesio-buccal, mid-buccal, disto-buccal, mesio-lingual, mid-lingual, and disto-lingual sits of all teeth using a CP-11 color-coded probe (Hu-Friedy Mfg. Co. LLC., Chicago, IL, USA) by two dental hygienists. Sites that bled upon gentle probing were recorded, and the proportions of sites with bleeding on probing (BOP) and the number of BOP-positive teeth were determined for each patient. For PPD, agreement to within 1 mm was 100% on preliminary calibration. Cohen’s kappa-index was 0.88. Periodontitis was diagnosed using panoramic X-rays according to the Japanese Society of Periodontology (2015) [34]. In brief, a dentist diagnosed periodontitis when clinical attachment loss or bone loss was evident [34]. Probed pocket depths or bone loss of >6 mm or >50%, 4–6 mm or 30%–50%, and <4 mm or <30%, respectively, were regarded as severe, moderate, and mild periodontitis [34], respectively if the patients had at least one site of the condition.

### 2.9. Questionnaire

Study personnel recorded medical histories, medications, and lifestyle information including alcohol consumption, smoking, toothbrushing frequency, and regularity of dental checkups. We also investigated personal self-efficacy based on scores on a self-efficacy scale for self-care (SESS) to confirm the effects of the six-step method [19,35].

### 2.10. General Status of Patients

Information about general and oral status was obtained from medical and dental records. Parameters were measured between the preoperative period and discharge. The extracted information included data about sex, age, body mass index (BMI), primary heart diseases (diseases of the circulatory system; International Statistical Classification of Diseases and Related Health Problems (ICD)-10), other diseases (ICD-10), medical history other than diseases of the circulatory system, type of surgery, surgical duration, duration of hospitalization, antibiotics, intubation, amount of bleeding during surgery, medications, number of days with fever ≥38.0 °C during one week, blood findings, and incidences of postoperative complications. 

### 2.11. Statistical Analysis

The unit of analysis was a patient. Data were statistically analyzed using SPSS software version 24.0 (Japan IBM Co., Tokyo, Japan). Primary and secondary variables were compared between the control and intervention groups using chi-square or t-tests. Outcomes between two groups were assessed using an analysis of covariance (ANCOVA) model that included surgical duration and type of surgery as covariates. Adjusted differences and 95% confidence intervals (CIs) were determined. Mean changes between the control and intervention groups determined by ANCOVA were compared using t-tests. Values with *p* < 0.05 were considered significantly different, whereas in cases of periodontal status and tongue status, the test with Bonferroni correction to control the false discovery rate was used (*p* < 0.05/4 and *p* < 0.05/2, respectively) [36].

The effect size was also assessed using Cohen’s d (t-test) [37]. Effect size is an indicator of the meaningfulness of a change in a health status measure. The small, medium and large effect sizes are d = 0.20, 0.50 and 0.80, respectively [37]. 

## 3. Results

### 3.1. Participants Information at Baseline

**Figure 1:**
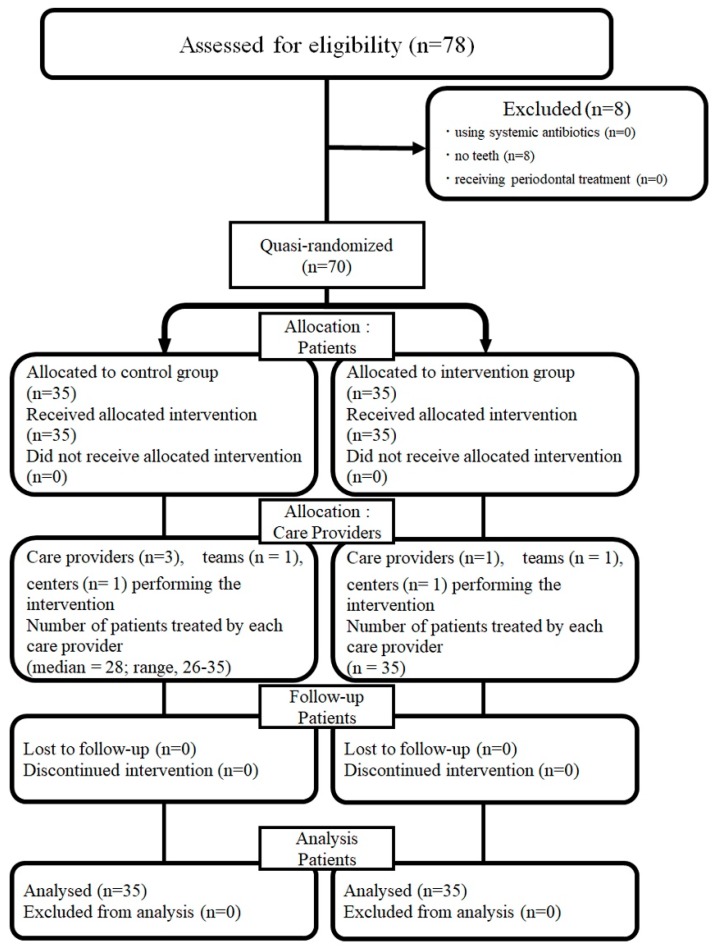
Flowchart of the study.

Figure 1 shows that among 78 screened individuals, 70 who were quasi-randomized between June 2016 and July 2017 completed the study (Figure 1).

Table 2 shows that the baseline characteristics were similar between the control and intervention groups, and that none of them significantly differed (*p* > 0.05). Furthermore, oral health behavior [frequency of toothbrushing (≥2/day), dental checkups (/year) and interdental brushing] at baseline was similar between the control and intervention groups [21 (60.0%) vs. 21 (60.0%), 1.1 ± 3.0 vs. 1.0 ± 2.4, 2 (5.7%) vs. 3 (8.6%), respectively], and none of them significantly differed (*p* > 0.05).

### 3.2. Participants Status at Postoperative

The numbers of postoperative oral hygiene care by dental hygienists were 4.5 ± 1.5 in the control group and 4.5 ± 1.4 in the intervention group. There was no significant difference in the number between the two groups (*p* = 0.806). Furthermore, there were no patients for whom self-care was difficult due to physical issues in the two groups.

Table 3 shows differences in postoperative systemic conditions and oral health behaviors between the control and intervention groups. Surgical duration, the amount of bleeding during surgery, length of stay in hospital, intensive care unit (ICU), and coronary care unit (CCU), duration of intubation, and number of postoperative days to ingestion did not significantly differ. On the other hand, the period (days) of postoperative AF was significantly shorter in the intervention than in the control group (*p* = 0.019). Furthermore, oral health behavior [frequency of toothbrushing (≥ 2/day) and interdental brushing] at discharge was improved compared to the baseline in the two groups and the number of patients who reported the use of interdental brushing in the intervention group was significantly larger than that in the control group. [33 (94.3%) vs. 19 (54.3%)] (*p* < 0.001).

Table 4 shows changes in the clinical parameters between the control and intervention groups. Significantly fewer oral bacteria were found on the tongue between baseline and discharge in the intervention, than the control group (*p* < 0.001; effect size was large). Decreases in the amount of tongue coating and PCR scores between baseline and two weeks after surgery and between baseline and discharge were significantly greater in the intervention, than the control group (*p* < 0.001; effect size was large). The decreases in mean PPD (mm), PPD ≥4 mm (%), and BOP (%) between baseline and discharge were also significantly greater in the intervention, than in the control group (*p* < 0.05; effect size was large except for mean PPD). Changes in SESS scores between baseline and discharge from hospital were more significant in the intervention, than in the control group (*p* = 0.001; effect size was large). Postoperative AF (day) persisted for significantly less time in the intervention, than the control group after adjustment (*p* = 0.019; effect size was medium).

The interventions in both groups were implemented according to the schedule. Oral hygiene instructions were of low risk, and no serious, study-related adverse events developed. None of the patients required generalized periodontal rescue therapy during the study. There were no patients who had a delay between randomization and the initiation of the intervention in both groups. Furthermore, outcomes did not change after the trial commenced.

## 4. Discussion

The intervention group had a significantly greater decrease from baseline in the numbers of oral bacteria on the tongue, scores of tongue coating, and PCR than the control group at discharge from hospital. Self-care as well as professional care is needed to improve oral hygiene. Behavioral science approaches such as the six-step method have been developed to improve self-efficacy, oral hygiene status, and periodontitis among dental outpatients [19,20]. To the best of our knowledge, this is the first study of the effects of the six-step method on the postoperative oral hygiene status of inpatients with periodontitis and heart diseases. These findings supported our hypothesis that the six-step method improves self-care and oral hygiene status among inpatients.

Periodontal parameters and self-efficacy improved more in the intervention, than the control group. The six-step method decreased %BOP and PPD and increased self-efficacy scores in a previous study of outpatients with periodontitis [20]. The present findings were similar to these, suggesting that the six-step method can help inpatients and outpatients with periodontitis to improve periodontal status and self-efficacy.

Postoperative AF persisted for a significantly shorter period (days) in the intervention, than the control group. Postoperative AF remains the most common complication of cardiac surgery [2,3,4]. Previous studies have associated local and systemic inflammation with AF [6,7] and others have suggested an association between AF and periodontitis [14,15]. Therefore, improving local inflammation or periodontitis using the six-step method might help to decrease the incidence or persistence of postoperative AF. However, further studies are required to investigate the mechanism of this relationship.

The amount of time required for oral hygiene instruction using the six-step method (approximately 15 min) was similar to that of routine oral hygiene instruction based on the guidelines of the Japanese insurance system. The two groups spent a similar amount of time in the ICU/CCU and hospital, and the six-step instructions may help to improve oral hygiene status and periodontal health without using extra time. However, few dental professionals work on inpatients during the perioperative period. Thus, instructions will require further modification to shorten hospital/ICU stays and resolve perioperative staff shortages.

The six-step method is a systematic means of helping patients make lifestyle changes [19,20,22]. It applies self-efficacy theory to educate patients based on the hypothesis that self-efficacy initially improves and subsequently causes behavioral change [38]. Self-efficacy significantly improved, and interdental brushing significantly increased in the intervention group. Therefore, the present results suggest that oral hygiene instruction enhanced self-efficacy and then promoted behavioral change in the intervention group. The concept was supported by our previous study [20]. 

The incidence of postoperative pneumonia was lower in the intervention, than the control group (2.9% vs. 11.4%), but the difference did not reach statistical significance. A previous review found that the incidence of postoperative pneumonia varied from 0.5%–28% [39]. Since medical workers at our hospital usually control pneumonia in postoperative patients, the incidence of pneumonia in the present control group was relatively low. Thus, we could not detect a significant difference between the groups because of the floor effect.

At baseline, mean PPD and %BOP in this study were within the previously published ranges of patients with periodontitis and heart diseases (mean PPD and %BOP, 2.2–4.2 mm and 38.4%–92.49%, respectively) [40,41,42]. On the other hand, in this study, PPD ≥4 mm (%) was 72.9 ± 23.3 (± SD) % at baseline, which was higher than a previous study among patients with stable coronary artery disease (64.6 ± 7.5%) [43]. Although participant age, country, sample size, and study design differed between the present and other investigations, caution is warranted in regard to the generalizability of the results.

The incidence of postoperative AF was 44.3% among the participants in the present study. The reported incidence of postoperative AF was 20%–50% [2,3,4], and that in the present study was within that range.

This study had several limitations. Longer follow-up studies are required because only a relatively short perioperative period was investigated. The short period may also have influenced the treatment response, particularly with respect to PPD reduction. Second, we enrolled a small patient cohort at a single center (selection bias), which might limit the ability to extrapolate our findings to the general population of inpatients. Therefore, further large-scale studies are needed to confirm our findings. Third, we could not investigate clinical attachment level because we had the limited time in a routine work of treatment. Fourth, we used quasi-randomization instead of randomization. We selected this method because we wanted to equalize the number of two groups, which was of advantage compared to simple randomization in case of a small number of participants. Finally, we did not investigate periodontal pathogenic species that may affect the results.

## 5. Conclusions

In conclusion, oral hygiene instruction using the six-step method significantly improved the numbers of oral bacteria on the tongue, periodontal status, and period of postoperative AF among inpatients with periodontitis and heart diseases.

## Figures and Tables

**Table 1:** Typical oral hygiene instruction in the control and intervention groups.

	Control	Intervention
Priority	Teaching skills	Counseling
Disclosing dental plaque accumulation	+	+
Technical guidance of brushing	Teaching how to use interdental brushes	Teaching how to use interdental brushes
Six-step method	−	Asking the reason why a patient did not use interdental brushes and getting the patient to identify the problem
STEP1:		
Identification of the problem in each patient		
STEP2:	−	Counseling the patient to use interdental brushes
Creation of commitment and confidence		
STEP3:	−	Instructing the patients to maintain the use of interdental brushes by self-monitoring
Increase awareness of behavior		
STEP4:	−	Setting an achievable plan, "use interdental brushes daily," for the patient
Development and implementation of an action plan		
STEP5:	−	Praising improvements in the oral hygiene status after the patient used interdental brushes daily
Evaluation of the plan		
STEP6:	−	Reinforcing the daily use of interdental brushes and encouraging the patient
Maintenance of change and prevention of relapse		

**Table 2:** Baseline characteristics of participants.

Parameter	Control (N = 35)	Intervention (N = 35)
Male	24 (68.6) *	24 (68.6)
Age (y)	70.9 ± 11.1	68.1 ± 11.4
BMI (kg/m^2^)	22.0 ± 4.1	22.5 ± 4.0
Habit		
Cigarette smoking	26 (74.3)	23 (65.7)
Alcohol consumption	13 (37.1)	15 (42.9)
Systemic conditions		
Primary heart disease		
Nonrheumatic mitral valve disorders	7 (20.0)	9 (25.7)
Nonrheumatic aortic valve disorders	5 (14.3)	7 (20.0)
Chronic ischemic heart disease	6 (17.1)	6 (17.1)
Atherosclerosis	8 (22.9)	4 (11.4)
Aortic aneurysm and dissection	2 (5.7)	5 (14.3)
Angina pectoris	3 (8.6)	2 (5.7)
Multiple valve diseases	3 (8.6)	1 (2.9)
Complications and vague descriptions of heart disease	1 (2.9)	0 (0.0)
Infection following procedure, not elsewhere classified	0 (0.0)	1 (2.9)
Other heart diseases	14 (40.0)	11 (31.4)
Medical history except for heart diseases (>2)	12 (34.3)	11 (31.4)
Medications for heart diseases) (>2)	27(77.1)	20 (57.1)
Medications for other diseases) (>2)	20 (57.1)	18 (51.4)
Type of surgery		
Aortic valve replacement	11 (31.4)	6 (17.1)
Cardiac valve annuloplasty	6 (17.1)	8 (22.9)
Vascular replacement	2 (5.71)	8 (22.9)
Bypass	16 (45.7)	12 (34.3)
Debridement	0 (0.0)	1 (2.9)
Oral status		
Number of teeth	16.8 ± 8.8	19.4 ± 8.8
DMFT	18.7 ± 7.7	17.9 ± 7.8
Periodontal status		
Mean PPD (mm)	4.5 ± 1.0	4.3 ± 1.0
PPD ≥4 mm (%)	76.4 ± 22.8	69.3 ± 23.6
Periodontitis severity		
Severe	32 (91.4)	32 (91.4)
Moderate	3 (8.6)	3 (8.6)
BOP (%)	70.6 ± 19.5	63.7 ± 21.2
PCR (%)	72.8 ± 17.5	73.3 ± 17.6
Tongue status		
Tongue coating score	1.1 ± 0.9	1.5 ± 1.0
Numbers of oral bacteria on tongue (×10^7^; cfu/mL)	3.8 ± 2.8	3.6 ± 2.8
SESS score	55.5 ± 13.3	54.6 ± 10.2

* Data are shown as means ± SD or N (%). BOP, bleeding on probing; DMFT, decayed missing and filled teeth; PCR, plaque control record; PPD, probed pocket depth; SESS, self-efficacy scale for self-care.

**Table 3:** Differences in postoperative conditions between control and intervention groups.

Parameter	Control (N = 35)	Intervention (N = 35)	*p* ^†^
Surgical factors			
Duration (min)	344 ± 162 *	347 ± 143	0.934
Blood loss (mL)	1208 ± 1518	1321 ± 1584	0.763
Duration of stays (days)			
Hospital	27.5 ± 14.0	29.5 ± 14.2	0.554
ICU	3.4 ± 3.3	3.1 ± 1.9	0.569
CCU	5.4 ± 5.8	3.6 ± 2.4	0.099
Medications			
Postoperative medications (>2)	33 (94.3)	30 (85.7)	0.615
Duration of antibiotics (days)	5.7 ± 7.2	4.5 ± 3.8	0.386
Acetaminophen (×10^2^ mg)	0.8 ± 1.1	0.8 ± 1.3	0.918
Flurbiprofen (mg)	0.3 ± 0.6	0.1 ± 0.4	0.166
Fentanyl (mg)	25.1 ± 12.3	28.7 ± 11.4	0.214
Remifentanil hydrochloride (mg)	2.8 ± 2.0	3.5 ± 2.0	0.152
PCA (mL)	8.9 ± 15.8	8.5 ± 13.3	0.908
Intubation period (days)	0.7 ± 2.0	0.5 ± 1.5	0.741
Postoperative duration to ingestion (days)	2.1 ± 3.4	2.4 ± 5.1	0.804
Number of days with fever ≥38.0 °C within one week	2.9 ± 2.9	2.1 ± 2.0	0.168
Blood test findings			
CRP POD 1 (mg/dL)	4.0 ± 3.0	3.8 ± 2.3	0.765
CRP POD 3 (mg/dL)	11.1 ± 6.8	11.1 ± 5.2	0.995
CRP POD 7 (mg/dL)	4.7 ± 2.7	4.7 ± 3.5	0.966
WBC POD 1 (×10^2^/μL)	94.1 ± 28.3	101.6 ± 26.0	0.248
WBC POD 3 (×10^2^/μL)	97.2 ± 37.4	142.4 ± 248.1	0.29
WBC POD 7 (×10^2^/μL)	77.6 ± 26.7	79.7 ± 23.4	0.716
Events			
Atelectasis	23 (65.7) ^†^	17 (48.6)	0.147
Postoperative pain	26 (74.3)	26 (74.3)	1
Pneumonia	4 (11.4)	1 (2.9)	0.164
Postoperative infection	6 (17.1)	4 (11.4)	0.495
Delirium	3 (8.6)	1 (2.9)	0.303
AF (day)	4.8 ± 7.6	1.5 ± 2.8	0.019
AF incidence (%)	19 (54.3)	12 (34.3)	0.092
Readmission	7 (20.0)	5 (14.3)	0.526

* Data are shown as means ± SD or n (%). ^†^ Chi-squared or t-tests. AF, atrial fibrillation; CCU, coronary care unit; CRP, C-reactive protein; ICU, intensive care unit; PCA, patient controlled analgesia; POD, postoperative day; WBC, white blood cell count.

**Table 4:** Differences in oral status, self-efficacy scale for self-care and atrial fibrillation between control and intervention groups.

Parameter	Control (N = 35)	Intervention (N = 35)	Adjusted Difference ^†^ (95% CI)	*p* ^‡^	Effect Size ^§^
**One week after baseline**					
PCR (%)	73.0 ± 22.5*	50.7 ± 19.4	−19.6 (−27.9 to −11.3)	<0.001	1.06
Tongue coating score	1.4 ± 1.0	1.6 ± 1.0	−0.1 (−0.7 to 0.3)	0.434	0.2
Numbers of oral bacteria on tongue (×10^7^) (cfu/mL)	2.4 ± 2.4	2.0 ± 1.7	−0.3 (−2.0 to 1.3)	0.71	0.19
**Two weeks after baseline**					
PCR (%)	63.5 ± 24.9	39.2 ± 22.1	−21.8 (−31.0 to −12.7)	<0.001	1.03
Tongue coating score	1.3 ± 1.0	0.7 ± 0.9	−1.1 (−1.6 to −0.5)	<0.001	0.63
**At discharge**					
Mean PPD (mm)	4.1 ± 0.9	3.6 ± 0.7	−0.3 (−0.5 to −0.1)	0.005	0.62
PPD ≥4 mm (%)	61.4 ± 26.8	39.4 ± 26.7	−14.4 (−21.9 to −6.9)	<0.001	1.16
BOP (%)	47.1 ± 22.6	27.6 ± 15.6	−11.4 (−19.0 to −3.90)	0.004	1.00
PCR (%)	62.5 ± 22.8	28.7 ± 16.9	−31.2 (−39.9 to −22.6)	<0.001	1.68
Tongue coating score	1.1 ± 1.0	0.3 ± 0.5	−1.2 (−1.7 to −0.6)	0.001	1.01
Numbers of oral bacteria on tongue (×10^7^) (cfu/mL)	3.0 ± 2.1	1.1 ± 0.9	−1.7 (−3.2 to −0.3)	0.02	1.18
SESS scores	52.7 ± 9.9	60.6 ± 10.1	8.7 (3.6 to 13.8)	0.001	0.99
AF (days)	4.8 ± 7.6	1.5 ± 2.5	−3.5 (−5.6 to −0.6)	0.019	0.58

* Means ± SD. †Adjusted for surgical duration and type of surgery. ^‡^ Changes in parameters between control and intervention groups based on t-tests after ANCOVA. ^§^ Cohen’s d. The small, medium and large effect sizes are d = 0.20, 0.50 and 0.80, respectively. AF, atrial fibrillation; BOP, bleeding on probing; CI, Confidence interval; PCR, plaque control record; PPD, probing pocket depth; SESS, self-efficacy scale for self-care.
